# Perceptions of use and value for different types of digital health solutions among people with type 1 and 2 diabetes in France

**DOI:** 10.1007/s00592-025-02564-6

**Published:** 2025-08-08

**Authors:** Norbert Hermanns, Paco Cerletti, Julie Laurent, Renza Scibilia, Sören Skovlund

**Affiliations:** 1 Research Institute of the Diabetes Academy Mergentheim (FIDAM), Johann Hammer Str. 24, 97980 Bad Mergentheim, Germany; 2 Department for Clinical Psychology and Psychotherapy of the University of Bamberg, Markusplatz 3, Bamberg, D-96047 Germany; 3 Roche Diagnostics International AG, Grenzacherstrasse 124, Basel, CH-4058 Switzerland; 4 Carenity, 1 rue de Stockholm, Paris, 75008 France; 5 Breakthrough T1D, 200 Vesey Street, New York, NY 10281 USA; 6 Arne Jacobsens Allé 7, DK-2300 Copenhagen, Denmark

**Keywords:** Clinical outcomes, Diabetes, Digital health solutions, Person-reported outcome measures, Quality of life

## Abstract

**Aims:**

This study examines the use, perceptions, and inequalities in access to Digital Health Solutions (DHS) among people with diabetes (PwD). It aims to identify factors influencing adoption and explore perceived benefits and barriers to using DHS, focusing on person-important outcomes such as physical health, mental burden, and access to care.

**Methods:**

The primary objective of this feasibility study was to assess the intervention acceptability, feasibility, and app usability. The secondary aim is to explore preliminary intervention effects.

**Methods:**

A cross-sectional online survey was conducted in France from April to July 2022. A total of 301 PwD (149 with type 1 diabetes [T1D], 152 with type 2 diabetes [T2D]) completed the study. The survey assessed the use of three DHS categories: information/education (DHS1), self-management support (DHS2), and data-sharing/collaborative care (DHS3). We used univariate and multivariate logistic regression to identify predictors of DHS use, including demographic, socioeconomic, psychological, and medical variables.

**Results:**

DHS1 was the most commonly used category, followed by DHS2 and DHS3. PwD with T1D were more likely to use multiple DHS. Type of diabetes and perceived health status were the strongest predictors of DHS use. Surprisingly, people in poorer health were less likely to use DHS despite potentially benefiting most from them. DHS-naïve individuals expected more benefits but reported greater concerns, especially about information overload and data security. These concerns were stronger than the perceived benefits. For example, concerns about data security reduced the likelihood of using DHS2 and DHS3 by up to 89%.

**Conclusion:**

The study highlights disparities in DHS adoption and the critical role of perceived barriers. Addressing these concerns—particularly among PwD in poorer health—and aligning DHS with outcomes that matter to patients may improve equitable adoption and diabetes care.

**Supplementary Information:**

The online version contains supplementary material available at 10.1007/s00592-025-02564-6.

## Introduction

Digital Health Solutions (DHS) hold great potential to transform the management and care of people with diabetes (PwD) and their families [1, 2]. By offering tools and resources tailored to different aspects of diabetes care, DHS aim to improve self-management, health outcomes and psychosocial aspects of living with diabetes [2–4]. One way to classify DHS is through a structured process that actively involves researchers, PwD, and representatives of diabetes advocacy organizations. This collaborative approach categorizes DHS into three primary categories, defined by their specific goals and functionalities [5]:

1. **DHS for Information and Education**: Platforms providing educational resources, videos, and forums to empower PwD with knowledge about diabetes management, lifestyle changes, and coping strategies (DHS1).
2. **DHS for Self-Management Support**: Technologies that utilize sensors to monitor and track health data such as glucose levels, physical activity, food intake and medications, enabling users to take control of and modify lifestyles important for diabetes management (DHS2).
3. **DHS for Data Sharing and Collaborative Care**: Tools that facilitate digital communication and data sharing between PwD and healthcare providers (HCP), allowing for timely interventions and adjustments to treatment plans without requiring frequent in-person visits. (DHS3) [5].

While DHS hold significant potential to improve outcomes in diabetes care, their use and perceived benefits appear to vary across the diabetes community [6]. Factors such as diabetes type, duration, health status, or psychological characteristics may influence adoption [7], but the extent and implications of these factors are not yet fully understood [8]. Differences in DHS use raise questions about whether certain groups are better positioned to access or benefit from these technologies, and whether barriers – such as concerns about data security, ease of use, or cost – deter some individuals. Identifying and addressing these potential barriers is critical to ensure that DHS contribute equitably to diabetes care [9, 10]. 

To truly evaluate the impact of DHS, we need a better understanding of PwD’s experiences, since current outcome measures focus mainly on clinical metrics like glucose levels or HbA1c and often ignore psychosocial and everyday effects that matter most to PwD [11, 12]. This highlights the need for DHS-specific person-reported outcome measures (PROMs) that capture key drivers of adoption, such as perceived improvements in self-management, reduced mental burden, and better access to care, alongside barriers like data overload or anxiety. PROMs developed with PwD input can offer a nuanced evaluation of DHS, shedding light on both benefits and potential drawbacks.

To address these knowledge gaps, this study seeks to explore who adopts digital health solutions (DHS) and which factors influence their use. It also examines how people with diabetes perceive the benefits and drawbacks of DHS on health, mental burden, and access to care.

## Methods

This paper reports findings from an observational cross-sectional cohort survey conducted in France. The methods of this observation study was already described in detail in a publication of Skovlund et al. [5]. However, in brief, some key methodological information.

### **Development of the survey questionnaire**

PwD co-designed the survey questionnaire and provided input to guide the sampling strategy and general study design [7, 9, 11, 13]. The survey questionnaire assesses PwD’s perceptions of use of DHS across the important outcome domains for each of three main DHS categories: (1) online/digital tools for information, education, support, and motivation; (2) personal health monitoring to support self-management; (3) digital and telehealth solutions for PWD-HCP data sharing and communication. The survey evaluates the perceived impacts of each DHS category on the core domains found previously to be important for PWD in relation to DHS: physical health, mental burden of treatment, ability to manage diabetes, access to care and support as well as potential disadvantages of DHS including information overload, being overwhelmed by diabetes, and anxiety about treatment failure and complications.

### **Eligibility criteria and sampling strategy**

All persons gave their informed consent prior to their inclusion in the study. The target sample consisted of adult PwD with an even split of Type 1 Diabetes (T1D) and Type 2 Diabetes (T2D), to ensure a sufficient statistical power to detect differences between both diabetes types. Purposive sampling was applied to maximize the comparability of the survey cohort with that of the general diabetes population in France.

###  Recruitment

This study was conducted in accordance with the ethical principles laid down in the Declaration of Helsinki. All participants received a detailed information sheet and provided informed, explicit, and documented consent prior to participation. The survey was fully anonymised. According to French regulations, ethics committee approval was not required, as the study did not involve any interventions, sensitive health data, or recruitment through healthcare professionals, and was classified as market research.This online survey was conducted from April to July 2022 in France. Data was collected through an online, cross-sectional survey. All participants were members of the Carenity platform and received a detailed information sheet and informed consent form (ICF) before participation. Consent was given electronically by clicking “Start” after reviewing the ICF, with a timestamp recorded as proof. This process ensured informed, explicit, and documented consent.

All approximately 13,000 adult members of the Carenity platform were informed about the study via email and invited to participate. Invitations were sent once per week, and participants also received private messages through the Carenity platform at the same frequency, resulting in two reminders per week. The recruitment period lasted for 6 weeks. The email open rate was approximately 18%, corresponding to around 2,340 individuals who opened the invitation. Of these, 580 persons initiated the survey (i.e., answered at least one question), and 301 completed the full questionnaire.

### Statistical and analytical methods

For the analysis of expectations to or experience with the use of DHS, we dichotomized the sample into people who were naïve to that particular DHS and experienced users, which included current and former users. This was based on the assessment that current and former users would both be responding from personal experience with use of the DHS in contrast to the DHS naïve persons.

We used a stepwise multinomial logistic regression to find out which factors predict how many types of DHS a person uses (none, one, two, or all three). To explore the role of perceived benefits and barriers, we also ran three separate stepwise logistic regression models—one for each DHS category (DHS1, DHS2, DHS3)—comparing users with non-users. In all models, type of diabetes was included as a predictor, regardless of its statistical significanceSPSS v29 and the multinom function in R were used.

### Sensitivity analysis

Because the Carenity sample differed in age distribution from the general diabetes population in France [14], we conducted a sensitivity analysis to address concerns about the sample’s representativeness. We applied statistical weighting using age data from the French National Health Insurance Agency (CNAM) [15] to adjust for this difference. All multivariable analyses were then repeated with the weighted sample to assess the robustness of our findings.

## Results

A total of 301 PwD, 149 people with T1D and 152 participants with T2D, completed the survey. Approximately the same share of women and men took part in the survey. Most participants were over 60 with diabetes for over ten years. More characteristics of the survey sample are described in Table 1.

**Table 1:** Sociodemographic data and characteristics of survey population

Characteristic	All *N* = 301	Type 1 Diabetes *N* = 149	Type 2 Diabetes *N* = 152	*P*
Age group:				
18–35 years, n (%)	17 (5.6)	15 (10.1)	2 (1.3)	
36–45 years, n (%)	22 (7.3)	16 (10.7)	6 (3.9)	
46–60 years, n (%)	10155 (51.5)	61 (40.9)	46 (30.3)	
> 60 years, n (%)	7 (35.5)	57 (38.3)	98 (64.5)	< 0.001
Diabetes duration				
< 1 year, n (%)	8 (2.7)	6 (4.0)	2 (1.3)	
1–4 years, n (%)	29 (9.6)	9 (6.0)	20 (13.2)	
4–9 years, n (%)	42 (14.0)	11 (7.4)	31 (20.4)	
≥ 10 years, n (%)	222 (73.8)	123 (82.6)	99 (65.1)	< 0.001
Sex				
Female, n (%)	159 (52.8)	86 (57.7)	73 (52.8)	
Male, n (%)	141 (46.8)	62 (41.6)	79 (52.0)	
No binary, n(%)	1 (0.3)	1 (0.7)	0 (0)	0.130
Years of education, mean (SD)	12.9 (± 2.4)	13.3 (± 2.2)	12.9 (± 2.4)	0.003
Paid 5- score, mean (SD)	11.0 (± 4.5)	11.3 (± 4.6)	12.9 (2.5)	0.169
Perceived health status				
Excellent	5 (1.7)	5 (3.4)	0 (0.0)	
Very good	33 (11.0)	22 (14.8)	11 (7.2)	
Good	183 (27.6)	76 (51.0)	71 (46.7)	
Intermediate	47 (48.8)	32(21.5)	51 (33.6)	
Poor	33 (11.0)	14 (9.4)	19 (12.5)	0.008
Number of Diabetes complications^a^				
None	212 (70.4)	116 (77.9)	96 (63.2)	
At least 1 complication	52 (17.3)	17 (11.4)	35 (23.0)	
> than 1 complication	37 (12.3)	16 (10.7)	218 (13.7)	0.012
Number of non-diabetes-related complications^b^				
None	190 (63.1)	110 (73.8)	80 (52.6)	
At least 1 complication	73 (24.3)	21 (14.1)	52 (34.2)	
> than 1 complication	38 (12.6)	18 (12.1)	20 (13.2)	< 0.001

^a^complications included were asthma, epilepsy, arthritis, sepsis, psoriasis, lupus, Covid, cancer, or thyroid gland; ^b^diabetes-related complications

were included: coronary heart disease, nephrology, neuropathy, retinopathy, or sexual dysfunction. PAID: Problem Areas in Diabetes Survey

**Fig. 1:**
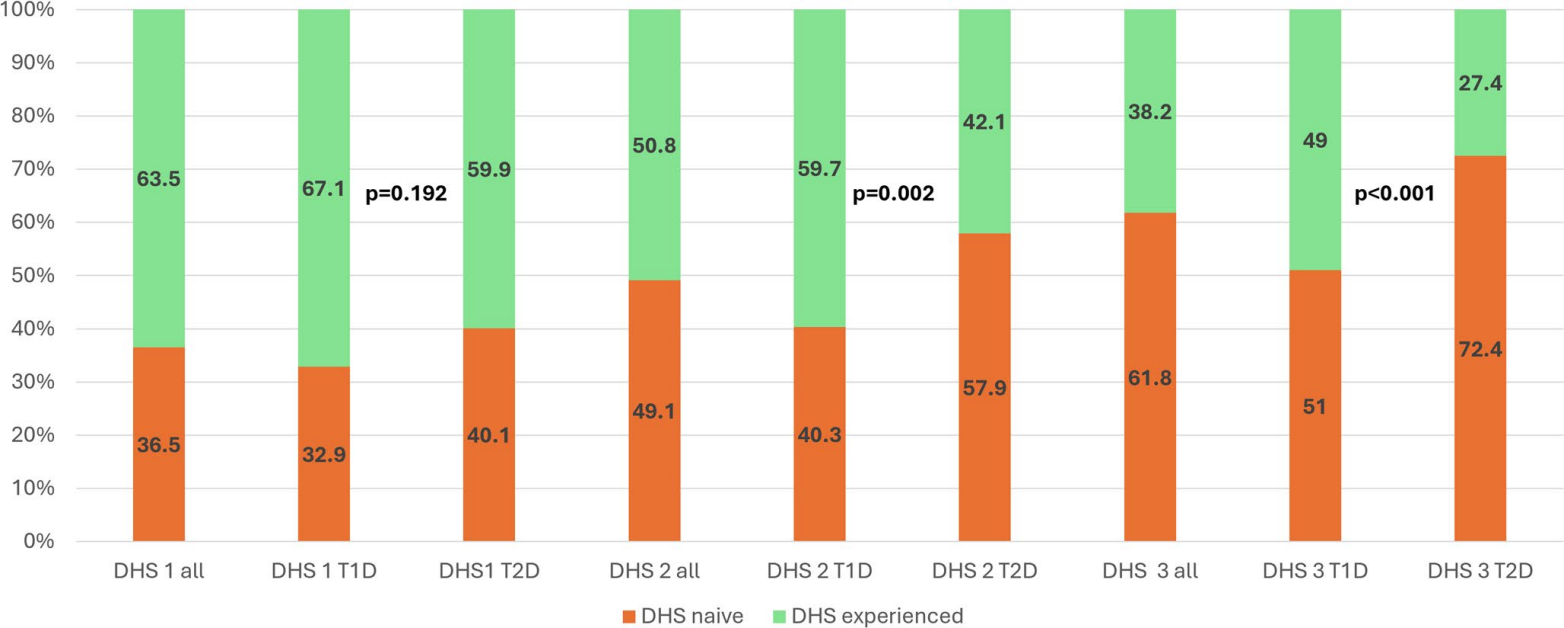
Proportion of DHS-naïve and experienced users across the three DHS categories. P-values indicate differences between participants with type 1 and type 2 diabetes for each DHS category

According to Fig. 1, DHS are most commonly used as digital information and educational resources (DHS1), followed by their application as sensors to track glucose levels (DHS2) and other health information as for example step count, heart rate, or sleep quality. The least used category is DHS3 for data-sharing and collaboration with Health Care Professionals (HCP). The use of DHS2 and DHS3 was significantly more frequent in people with type 1 diabetes.

### Drivers of DHS adoption

**Fig. 2:**
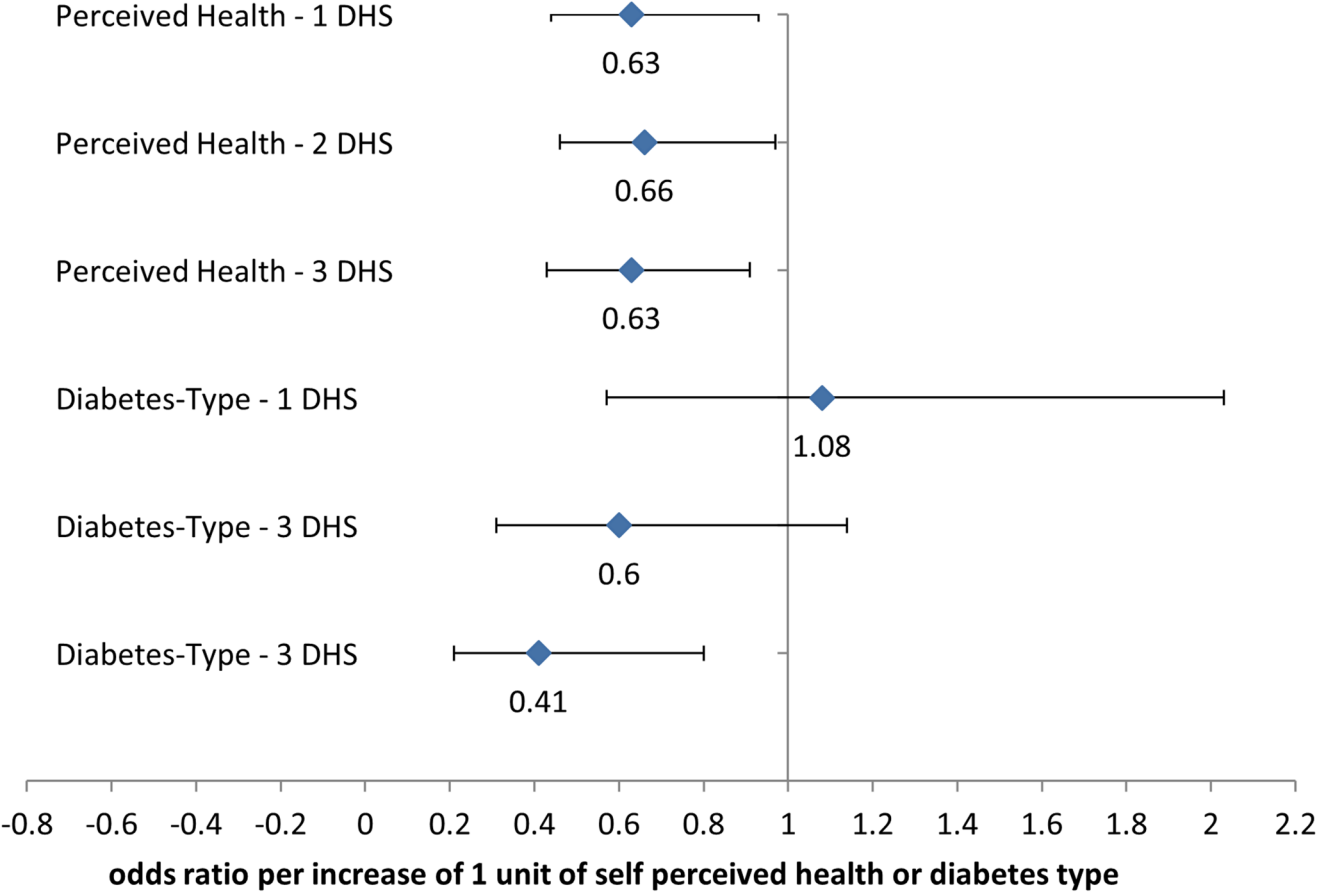
Stepwise multinomial regression showing predictors of DHS adoption (0–3 DHS used). Included variables: diabetes type, gender, age, duration, perceived health status, complications, education, and distress scores. Only predictors retained in the final model are shown

The results of the stepwise multinomial regression analysis (Fig. 2) show a somewhat counterintuitive pattern: the worse participants rated their health, the less likely they were to use any DHS. This is surprising, as those in poorer health might be expected to benefit most from digital health support. In contrast, people with type 1 diabetes were significantly more likely to use all three DHS categories. Other variables—such as age, education, psychological distress, or the presence of diabetes complications—were excluded from the final model, as they did not show a significant association with DHS use or access (see Supplementary Table 1).

### Perceived benefits and barriers of DHS adoption

Table 2 shows the descriptive survey responses for DHS naïve and experienced respondents for DHS1, DHS2 and DHS3. The most commonly benefit, reported by more than half of both naive and experienced respondents, was improved ability to self-manage diabetes for both DHS2 and DHS3, and improved access to diabetes care and support for DHS1. The most frequently disadvantage of DHS for naïve and experienced users was “making diabetes take up too much space in daily lives”, followed by “feeling overwhelmed by the demands of diabetes.”

**Table 2:** Perceptions of impacts of DHS categories 2 and 3 among PwD with or without (naïve) previous or current experience with the DHS (*N* = 301)

	DHS1: DHS for Information and Education with % agree		Chi Square *p* ^a^	DHS2: Personal health monitoring for self-management with % agree		Chi Square *p* ^a^	DHS3: DHS for HCP collaboration % agree		Chi Square *p*^a^
	Naïve *N* = 110	Experienced *N* = 191		Naïve *N* = 148	Experienced *N* = 153		Naïve *N* = 186	Experienced *N* = 115	
Core benefit outcome domains									
“Improve people’s physical health” respectively “It is improving/has improved my physical health”	57.3%	30.9%	**< 0.001**	64.9%^2^	41.2%^b^	**< 0.001**	57.5%^2^	38.3%^b^	**< 0.001**
“Reduce the mental burden of managing diabetes in the day-to-day” respectively ”It is reducing/has reduced the mental burden of managing diabetes in the day-to-day”	60,9%	30.9%	**< 0.001**	60,8%	37.9%	**< 0.001**	47.3%	42.6%	0.426
“Improve people’s ability to manage their diabetes (e.g. diet, exercise, blood glucose testing, taking medication, etc.)” respectively “It is improving/has improved my ability to manage my diabetes (e.g., diet, exercise, BG testing, medication)”	65.5%	45.0%	**< 0.001**	71.6%	54.9%	**0.003**	68.3%	54.8%	**0.018**
“Improve people’s access to diabetes care and support“ respectively “It is improving/has improved the care and support I receive(d) for my diabetes” respectively	70.0%	30.4%	**< 0.001**	N.A.^3^	N.A.^c^	N.A.^c^	66.1%	49.6%	**0.004**
“Reduce the cost of diabetes (e.g., through savings on transport and other costs)” respectively “It is reducing/has reduced the cost of diabetes care (e.g., through savings on transport and other costs)” respectively	N.A.	N.A.	N.A.	N.A.	N.A.	N.A.	45.7%	28.7%	**0.003**
“Improve the way diabetes care is focused on people’s priorities and issues” respectively “It is improving/has improved the way my diabetes care is focused on my priorities and issues”	N.A.	N.A.	N.A.	N.A.	N.A.	N.A.	50.5%	40.9%	0.102
“Improve people’s involvement in decisions about diabetes treatment“ respectively “It is improving/has improved my involvement in decisions about my diabetes treatment”	N.A.	N.A.	N.A.	N.A.	N.A.	N.A.	65.6%	48.7%	**0.004**
“Facilitate the coordination of people’s doctors for all their pathologies” respectively “It facilitates/facilitated the coordination of all my doctors for all my pathologies”	N.A.	N.A.	N.A.	N.A.	N.A.	N.A.	68.8%	50.4%	**0.001**
“Negative impacts”									
“Make people feel overwhelmed by too much information” respectively “It makes/has made me feel overwhelmed by too much information”	35.5%	14.7%	**< 0.001**	40.5%	14.4%	**< 0.001**	38.7%	10.4%	**< 0.001**
“Make people feel overwhelmed by the demands of diabetes” respectively “It makes/has made me feel overwhelmed by the demands of diabetes”	35.5%	35.1%	0.948	44.6%	34.6%	0.077	38.7%	23.5%	**0.006**
“Make people feel that they are failing in their diabetes management” respectively “It makes/has made me feel that I am failing in my diabetes management respectively”	28.2%	28.3%	0.987	35.1%	27.5%	0.150	34.4%	20.0%	**0.007**
“Make people feel that diabetes takes up too much space in their daily lives” respectively “It makes/has made diabetes take up too much space in my daily life”	34.5%	45.5%	0.062	46.6%	42.5%	0.470	43.5%	30.4%	**0.023**
It makes/has made me feel stressed about not knowing what health information to trust	45.5%	0%	**< 0.001**	N.A.	N.A.	N.A.	N.A.	N.A.	N.A.
It makes/has made me anxious because of alarming information about diabetes complications	47.3%	38.77´%	0.149	N.A.	N.A.	N.A.	N.A.	N.A.	N.A.
“Make people feel worried that their personal health data is not properly secured” respectively “It makes/made me worried that my personal health data is not properly secured”	N.A.	N.A.	N.A.	38.5%	13.1%	**< 0.001**	39.2%	9.6%	**< 0.001**
“Make people feel worried that they don’t record their personal health data properly (data entry error, etc.)” respectively “It makes/made me worried that I don’t record my personal health data properly (data entry error, etc.)” respectively”	N.A.	N.A.	N.A.	37.2%	5.9%	**< 0.001**	N.A. ^3^	N.A. ^3^	N.A. ^3^
“Make people feel worried that their health data is used to judge them” respectively “I makes/made me worried that my health data is being used to judge me”	N.A.	N.A.		N.A.	N.A.	N.A.	38.7%	7.8%	**< 0.001**
“Make people’s relationships with their diabetes care team more impersonal” respectively “It makes/made my relationship with my diabetes care team more impersonal”	N.A.	N.A.		N.A.	N.A.	N.A.	29.6%	6.1%	**< 0.001**

^a^p-values are results of chi-square tests; ^2^All percentage results are column percentages, i.e. the percentages refer to the proportion of participants in the respective groups (naive vs. experienced DHD users) who agree with the statement in the respective first column; ^c^N.A. not applicable because this statement was not presented to the users of DHS2 respectively DHS3

DHS1-naïve users generally had higher expectations than experienced users regarding improvements in physical health, reduced mental burden, greater self-management, and better access to care. The most notable difference was that DHS1-naïve users were more concerned about identifying trustworthy health information.

For DHS2, expectations that use of DHS2 could improve physical health, reduce the burden of diabetes management, and improve the person’s ability to better manage their diabetes was significantly higher among naïve DHS users than among experienced users (Table 2). There were no significant differences in the responses of DHS2 naïve and experienced users to expected or experienced negative impacts relating to diabetes-related emotional stress, specifically; feeling that DHS makes diabetes take up too much space, feeling overwhelmed by the demands of diabetes and feeling one is failing their diabetes management. Experienced DHS users reported fewer disadvantages, such as information overload or data security concerns. Worries about health data security and recording were very rare in experienced users of DHS2 and 3 to 6 times more common in the naïve group. This may suggests that these particular concerns among naïve do not reflect the views of most experienced DHS users (Table 2).

For DHS3, naïve and experienced users reported the same levels of experienced or expected benefits in terms of impact on mental burden and focus of care on their personal priorities. In contrast, for benefits relating to improved physical health, better ability to manage diabetes, better access to care, lower costs, greater involvement in treatment decisions or better coordinated diabetes care, naïve participants expected significantly more benefits than experienced DHS3 users reported (Table 2). DHS3 naïve people had significantly more pessimistic views about all the potential disadvantages of DHS3 compared to experienced DHS3 users, i.e. being overwhelmed by too much information and diabetes demands, having to deal with diabetes care failures more often, giving diabetes care too much space in their lives, data security issues, worrying about being judged based on their health data, or expecting a more impersonal relationship with their diabetes team (Table 3).

For both DHS2 and DHS3, naïve persons expressed a 3 to 4 times greater concern about being “overwhelmed by too much information” and “worries about personal data security” compared to experienced users.

**Fig. 3:**
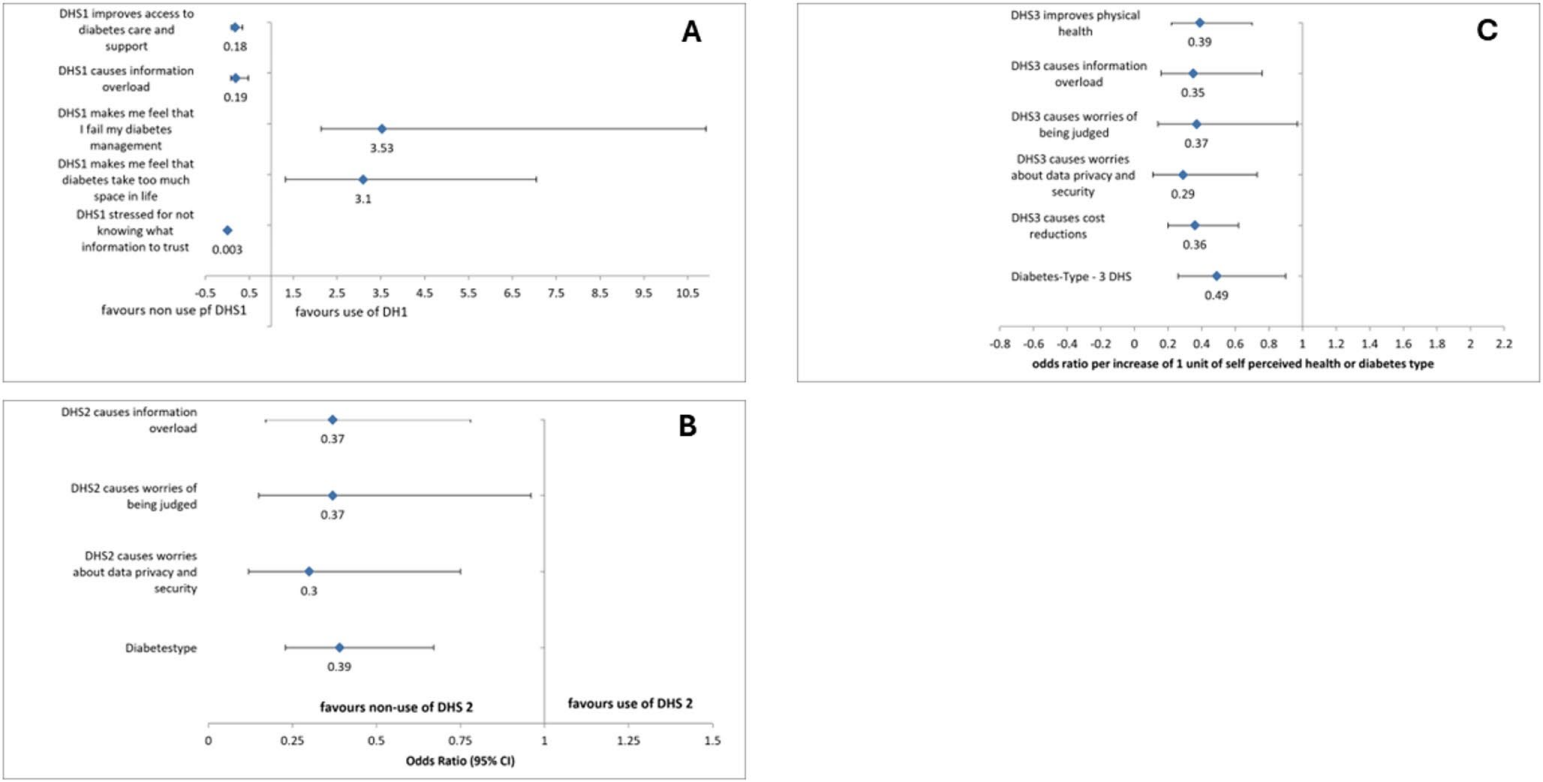
A, B, C Stepwise logistic regressions identifying factors associated with use (vs. non-use) of DHS1 (A), DHS2 (B), and DHS3 (C). Models include sociodemographic and attitudinal predictors (e.g., perceived benefits, barriers, and diabetes type). Odds ratios and 95% confidence intervals are shown. Only variables retained in the final models are displayed. Full item descriptions are provided in Table 2

Stepwise multivariate regression analysis was used to assess which of the experiences, shown in Table 2, were independently and significantly associated with the current or previous use of DHS1, DHS2 and DHS3 (Fig. 3A and C). While the perceived advantages and disadvantages were balanced in the univariate analysis, the multivariate stepwise analysis showed a preponderance of perceived disadvantages. For DHS1, the only significant benefit linked to its use was improved access to diabetes care and social support. Key barriers included fears of information overload and difficulty identifying trustworthy information. Interestingly, feelings of failure in diabetes management and the perception of diabetes as intrusive were positively associated with DHS1 use. For DHS2, the only expected benefit associated with use was improved physical health. Interestingly higher expectations that DHS2 improved physical health were associated with a 74% reduced likelihood of using DHS2 (odds ratio 0.26 95% CI (CI) 0.15–0.44, *p* < 0.05). Fear of information overload or concerns about data security independently reduced the likelihood of using DHS2 by 57% (odds ratio 0.43 CI 0.22–0.82, *p* < 0.05) and 89% (odds ratio 0.11 CI 0.05–0.27, *p* < 0.05). respectively. The latter barrier appeared to be the greatest. Having T2D reduced the likelihood of using DHS2 by 54% (odds ratio 0.46 CI 0.27–0.78, *p* < 0.001). The second stepwise regression for DHS3 use showed a similar picture. High expectations of improved physical health reduced the likelihood of using DHS3 by 68% (odds ratio 0.32 CI 0.18–0.56, *p* < 0.05). Fear of information overload or being judged by health data reduced this likelihood by 63% ((odds ratio 0.37 CI 0.17–0.78, *p* < 0.05 respectively (odds ratio 0.37 CI 0.15–0.96, *p* < 0.05)., concerns about data security and having T2D reduced the likelihood of using DHS3 by 70% (odds ratio 0.30 CI 0.12–0.75, *p* < 0.05) and 61% (odds ratio 0.39 CI 0.23–0.6, *p* < 0.05) respectively.

### Sensitivity analysis using weighted sample

We repeated analyses using the age-weighted sample. The results remained largely consistent with the above reported results, indicating that our findings are not substantially biased by the age distribution of the sample. Results are shown in Supplementary Tables 1 and Supplementary Fig. 2a–c.

## Discussion

This study examines the use, perceptions and inequalities in access to digital health solutions (DHS) among people with diabetes (PwD). Key findings show that patterns of use differ by type of diabetes: while information- and education-focused possibilities (DHS1) were used equally by people with type 1 and type 2 diabetes, self-management support (DHS2) and data-sharing DHS (DHS3) were significantly more common among people with type 1 diabetes. No significant sociodemographic, psychological or medical differences (e.g. age, gender, education or complications) were observed, although PwD with poorer self-reported health were less likely to use them, suggesting that health-related barriers outweigh structural inequalities. Naïve users of DHS generally expected greater health benefits from DHS2 and DHS3 than experienced users, but reported more perceived barriers, including concerns about information overload, data security and judgement.

Privacy concerns and fear of judgment based on shared health data mirror broader debates around digital trust and the psychosocial impact of digital surveillance in chronic disease management [16]. These apprehensions, particularly among DHS-naïve individuals, may reflect anticipated stigma and reduced autonomy, thereby inhibiting engagement. Addressing these trust barriers is critical for equitable adoption.”

For DHS 1 adoption there was also a preponderance of perceived barriers like fear of information overload, difficulties to identify trustfully health information over perceived benefits. The lack of significant differences in the use of information and education-focused DHS1 aligns with prior findings underscoring the universal need for education in diabetes management [17, 18]. However, the greater adoption of DHS2 and DHS3 among people with type 1 diabetes likely reflects the higher demands of their condition, including continuous glucose monitoring, insulin adjustments, and use of advanced diabetes technology. Age-related adoption patterns may also explain higher DHS2 and DHS3 use in T1D. Additionally, earlier adoption and greater integration of these DHS into type 1 diabetes care likely play a role.

Contrary to earlier studies [19, 20], this study found no significant gender differences or effects of education as a proxy for health literacy in DHS adoption [21, 22]. However, PwD with poorer self-reported health were less likely to use DHS. Barriers may include low digital or health literacy and physical or cognitive limitations.

DHS-naïve PwD expected significantly greater health benefits from DHS1, DHS2 and DHS3. Higher agreement on benefits was linked to DHS naïveté. This suggests that experienced users may have a more realistic view of the benefits of DHS, whereas DHS naïve individuals seem to have overly optimistic expectations. Consistent with the Technology Acceptance Model [23], such unmet expectations could lead to disappointment and eventual disengagement from technology use [24, 25].

The stepwise multivariate logistic regression highlighted the critical role of barriers in DHS adoption. Concerns such as information overload, data security, and privacy were more influential than perceived benefits. Moreover, fears of being judged based on shared health data may reflect experiences of diabetes-related stigma [13, 26], further discouraging adoption.

Our findings align with key components of the Technology Acceptance Model (TAM) [27] and the Unified Theory of Acceptance and Use of Technology (UTAUT) [28], which posit that perceived usefulness, ease of use and social influence are central to the adoption of technology. In our study, we observed that perceived barriers, such as information overload or data security concerns, reduce perceived ease of use, while unmet expectations among inexperienced users reflect discrepancies in perceived usefulness. These insights highlight the importance of trust and expectancy management for achieving long-term engagement with the DHS.

Recent systematic reviews and cross-national studies have shown that DHS adoption varies widely across countries, shaped by health system integration, digital literacy, and trust in technology providers [19, 20]. Our findings from France echo global concerns regarding data security and the digital divide, particularly among older adults and people in poorer health. These disparities highlight the need for tailored interventions that account for structural and cultural differences.

Several limitations should be considered. First, the cross-sectional design limits causal inference. Associations were found, but temporal relationships remain unclear. Secondly, we acknowledge that the 50% proportion of respondents with type 1 diabetes does not reflect the broader diabetes population. The purposive sampling design, with equal representation of T1D and T2D, facilitated subgroup comparisons but limits real-world generalizability. Moreover, recruiting from a self-selected online community (Carenity) may have introduced bias toward digitally literate or engaged individuals, underrepresenting PwD with low digital access or interest.

Third, the low overall response rate (2.3%) raises concerns about potential selection bias. Respondents may differ in attitudes, tech access, or behaviours, limiting generalizability. For example, individuals more familiar with digital health solutions (DHS) may be overrepresented, while those less engaged with technology may be underrepresented. Additionally, the Carenity sample was younger than the general population with diabetes in France [14], potentially reinforcing this bias. Recruitment was capped at 150 participants per diabetes type, then closed. As a result, the low overall participation rate may underestimate the true response rate among those who received and opened the invitation. Moreover, a sensitivity analysis using age-weighted data suggests that the main results were not materially affected by age-related differences in the sample.

Forth, the reliance on self-reported data introduces biases such as recall and social desirability bias, which may bias reported health status, DHS use and perceptions. In addition, cultural, health system or regional differences may influence DHS uptake, further limiting generalizability beyond this context.

Fifth, while stepwise regression facilitates exploratory model building, it may increase risk of overfitting. Our use of this method aimed to identify dominant predictors of DHS use while recognizing its limitations in inferential generalizability.

Future research should also address barriers such as data security concerns and stigma, and test interventions to improve engagement, particularly among those in poorer health. Research in other chronic conditions could broaden insights.

This study identifies key factors influencing DHS adoption in diabetes care. Addressing barriers and tailoring solutions can improve equitable access and outcomes. Future research should use longitudinal designs to clarify causal relationships between health status, technology perceptions and DHS use. Larger, more diverse samples are needed to reduce selection bias and include underrepresented groups, such as those with limited digital literacy. Combining objective measures of DHS use with self-reported data could improve accuracy. Research should also address barriers such as data security concerns and stigma, and test interventions to improve engagement, particularly among those in poorer health. Extending the research to other chronic conditions could provide broader insights.

In summary, this study identifies key factors influencing DHS adoption and provides important insights into the use of digital health for diabetes management. Addressing barriers and tailoring solutions can improve equitable access and improve health outcomes in diabetes care.

## Electronic supplementary material

Below is the link to the electronic supplementary material.

Supplementary Material 1

## Data Availability

The following data can be shared: individual participant data underlying the results reported in this article after de-identification (text, tables, figures, and appendices). Additionally, the study protocol can be made available. Data sharing can commence immediately following publication and continue until 10 years of publication. The data will be shared with researchers who provide a methodologically sound proposal. The sharing of the data needs to fulfil the purpose of achieving the aims of the approved proposal. Proposals should be directed to hermanns@fidam.de. To gain access to the data, the requestors will need to sign a data access agreement.
