# Supplementary material for: Perceptions of use and value for different types of digital health solutions among people with type 1 and 2 diabetes in France
Source: Acta Diabetol. 2025 Aug 8;63(1):37–47. doi: 10.1007/s00592-025-02564-6 (PMC12847089; doi:10.1007/s00592-025-02564-6)
Supplement: Supplementary file 1 — Supplementary Material 1 [file 592_2025_2564_MOESM1_ESM.docx]

**Supplementary Table 1**: Odds Ratios and Confidence Intervals by Number of DHS of the whole model predicting number of DHS use

| Predictor | Variable | Odds Ratio | CI Lower | CI Upper | P-Value |
| --- | --- | --- | --- | --- | --- |
| Number of DHS = 1 | Diabetes duration | 0.987299 | 0.909058 | 1.07227 | 0.761549 |
| Number of DHS = 2 | Diabetes duration | 1.03016 | 0.93983 | 1.12916 | 0.525704 |
| Number of DHS = 3 | Diabetes duration | 1.06987 | 0.968752 | 1.18155 | 0.182438 |
| Number of DHS = 1 | Intercept | 2.86854 | 0.140688 | 58.4879 | 0.493308 |
| Number of DHS = 2 | Intercept | 1.066 | 0.0450362 | 25.232 | 0.968421 |
| Number of DHS = 3 | Intercept | 4.0403 | 0.168894 | 96.6526 | 0.38867 |
| Number of DHS = 1 | Age-group | 0.989673 | 0.959377 | 1.02093 | 0.512865 |
| Number of DHS = 2 | Age-group | 0.992804 | 0.962218 | 1.02436 | 0.651042 |
| Number of DHS = 3 | Age-group | 0.986596 | 0.957194 | 1.0169 | 0.381987 |
| Number of DHS = 1 | Diabetes-complications | 0.623993 | 0.362259 | 1.07483 | 0.0891504 |
| Number of DHS = 2 | Diabetes-complications | 0.931394 | 0.57262 | 1.51496 | 0.774601 |
| Number of DHS = 3 | Diabetes-complications | 0.923667 | 0.555713 | 1.53526 | 0.759377 |
| Number of DHS = 1 | Diabetestype | 1.11084 | 0.547211 | 2.25502 | 0.77106 |
| Number of DHS = 2 | Diabetestype | 0.650571 | 0.319114 | 1.3263 | 0.236829 |
| Number of DHS = 3 | Diabetestype | 0.499781 | 0.241614 | 1.0338 | 0.061435 |
| Number of DHS = 1 | Health-score | 0.7577 | 0.494417 | 1.16119 | 0.202702 |
| Number of DHS = 2 | Health-score | 0.667759 | 0.434129 | 1.02712 | 0.0660327 |
| Number of DHS = 3 | Health-score | 0.631568 | 0.411365 | 0.969645 | 0.0356477 |
| Number of DHS = 1 | paid5_sum | 0.977698 | 0.905589 | 1.05555 | 0.563943 |
| Number of DHS = 2 | paid5_sum | 1.0301 | 0.950292 | 1.11661 | 0.471054 |
| Number of DHS = 3 | paid5_sum | 1.0057 | 0.928387 | 1.08946 | 0.889208 |
| Number of DHS = 1 | sex | 1.31673 | 0.687331 | 2.52247 | 0.406782 |
| Number of DHS = 2 | sex | 1.48739 | 0.772893 | 2.86239 | 0.234561 |
| Number of DHS = 3 | sex | 0.957602 | 0.492896 | 1.86043 | 0.898263 |
| Number of DHS = 1 | Year of education | 0.979126 | 0.859116 | 1.1159 | 0.75185 |
| Number of DHS = 2 | Year of education | 1.02279 | 0.893567 | 1.17069 | 0.743706 |
| Number of DHS = 3 | Year of education | 1.01955 | 0.888277 | 1.17023 | 0.783038 |

**Supplementary Table 2**. Perceptions of impacts of DHS categories 1, 2 and 3 among PwD with or without (naïve) previous or current experience with the DHS (N=301) weighted by the age distribution of the national health insurance in France (Caisse nationale de l’Assurance Maladie)

|  | **DHS1: DHS for Information and Education with % agree** | | **Chi Square**  **p^a^** | **DHS2: Personal health monitoring for self-management with  % agree** | | **Chi Square**  **p^a^** | **DHS3: DHS for HCP collaboration % agree** | | **Chi Square p^a^** |
| --- | --- | --- | --- | --- | --- | --- | --- | --- | --- |
|  | Naïve  N=110 | Experienced  N=191 |  | Naïve  N=148 | Experienced  N=153 |  | Naïve  N=186 | Experienced  N=115 |  |
| **Core benefit outcome domains** |  | | | | | | | | |
| “Improve people’s physical health” respectively “It is improving/has improved my physical health” | 61.1% | 31.1% | **<0.001** | 64.9%^2^ | 39.1%^b^ | **<0.001** | 57.3%^2^ | 37.5%^b^ | **<0.001** |
| “Reduce the mental burden of managing diabetes in the day-to-day” respectively ”It is reducing/has reduced the mental burden of managing diabetes in the day-to-day” | 62.4% | 30.2% | **<0.001** | 59.9% | 35.1% | **<0.001** | 44.6% | 41.6% | 0.632 |
| “Improve people’s ability to manage their diabetes (e.g. diet, exercise, blood glucose testing, taking medication, etc.)” respectively “It is improving/has improved my ability to manage my diabetes (e.g., diet, exercise, BG testing, medication)” | 73.5% | 53.0% | **<0.001** | 71.6% | 54.9% | **0.003** | 67.6% | 55.8% | **0.040** |
| “Improve people’s access to diabetes care and support“ respectively “It is improving/has improved the care and support I receive(d) for my diabetes” respectively | 68.8% | 31.1% | **<0.001** | N.A.^3^ | N.A.^c^ | N.A.^c^ | 65.4% | 50.4% | **0.004** |
| “Reduce the cost of diabetes (e.g., through savings on transport and other costs)” respectively “It is reducing/has reduced the cost of diabetes care (e.g., through savings on transport and other costs)” respectively | N.A. | N.A. | N.A. | N.A. | N.A. | N.A. | 45.9% | 29.2% | **0.004** |
| “Improve the way diabetes care is focused on people’s priorities and issues” respectively “It is improving/has improved the way my diabetes care is focused on my priorities and issues” | N.A. | N.A. | N.A. | N.A. | N.A. | N.A. | 50.3% | 40.4% | 0.095 |
| “Improve people’s involvement in decisions about diabetes treatment“ respectively “It is improving/has improved my involvement in decisions about my diabetes treatment” | N.A. | N.A. | N.A. | N.A. | N.A. | N.A. | 65.1% | 48.7% | **0.004** |
| “Facilitate the coordination of people’s doctors for all their pathologies” respectively “It facilitates/facilitated the coordination of all my doctors for all my pathologies” | N.A. | N.A. | N.A. | N.A. | N.A. | N.A. | 67.6% | 49.6% | **0.002** |
| **“Negative impacts”** |  |  |  |  |  |  |  |  |  |
| “Make people feel overwhelmed by too much information” respectively “It makes/has made me feel overwhelmed by too much information” | 36.1% | 15.3% | <0.001 | 41.9% | 14.6% | **<0.001** | 39.5% | 10.6% | **<.001** |
| “Make people feel overwhelmed by the demands of diabetes” respectively “It makes/has made me feel overwhelmed by the demands of diabetes” | 43.5% | 34.4% | 0.123 | 44.6% | 34.6% | 0.077 | 39.5% | 21.2% | **0.001** |
| “Make people feel that they are failing in their diabetes management” respectively “It makes/has made me feel that I am failing in my diabetes management respectively” | 33.3% | 26.5% | 0.208 | 35.1% | 27.5% | 0.150 | 33.0% | 19.5% | **0.012** |
| “Make people feel that diabetes takes up too much space in their daily lives” respectively “It makes/has made diabetes take up too much space in my daily life” | 46.3% | 42.1% | 0.487 | 46.6% | 42.5% | 0.470 | 44.3% | 30.1% | **0.014** |
| It makes/has made me feel stressed about not knowing what health information to trust | 45.5% | 0.5% | **<0.001** | N.A. | N.A. | N.A. | N.A. | N.A. | N.A. |
| It makes/has made me anxious because of alarming information about diabetes complications | 49.5% | 39.5´% | 0.115 | N.A. | N.A. | N.A. | N.A. | N.A. | N.A. |
| “Make people feel worried that their personal health data is not properly secured” respectively “It makes/made me worried that my personal health data is not properly secured” | N.A. | N.A. | <0.001 | 40.3% | 8.81.3% | **<0.001** | 40.3% | 8.8% | **<0.001** |
| “Make people feel worried that they don’t record their personal health data properly (data entry error, etc.)” respectively “It makes/made me worried that I don’t record my personal health data properly (data entry error, etc.)” respectively” | N.A. | N.A. |  | 37.4% | 6.0% | **<0.001** | N.A. ^3^ | N.A. ^3^ | N.A. ^3^ |
| “Make people feel worried that their health data is used to judge them” respectively “I makes/made me worried that my health data is being used to judge me” | N.A. | N.A. |  | N.A. | N.A. | N.A. | 39.5% | 6.2% | **<0.001** |
| “Make people’s relationships with their diabetes care team more impersonal” respectively “It makes/made my relationship with my diabetes care team more impersonal” | N.A. | N.A. |  | N.A. | N.A. | N.A. | 29.2% | 6.2% | **<0.001** |

^a^p-values are results of chi-square tests; ^2^All percentage results are column percentages, i.e. the percentages refer to the proportion of participants in the respective groups (naive vs. experienced DHD users) who agree with the statement in the respective first column; ^c^N.A. not applicable because this statement was not presented to the users of DHS2 respectively DHS3

**Supplementary Figure 1**. Age-weighted results of a stepwise multinomial regression analysis with the number of adopted digital health solutions (DHS) as the dependent variable. Independent variables included gender, diabetes type, diabetes duration, age group, perceived health status, diabetes distress scores, diabetes complications, and years of education. The reference category is non-use of DHS (0 DHS). Only variables retained in the final model are shown; variables not displayed were excluded during stepwise selection.


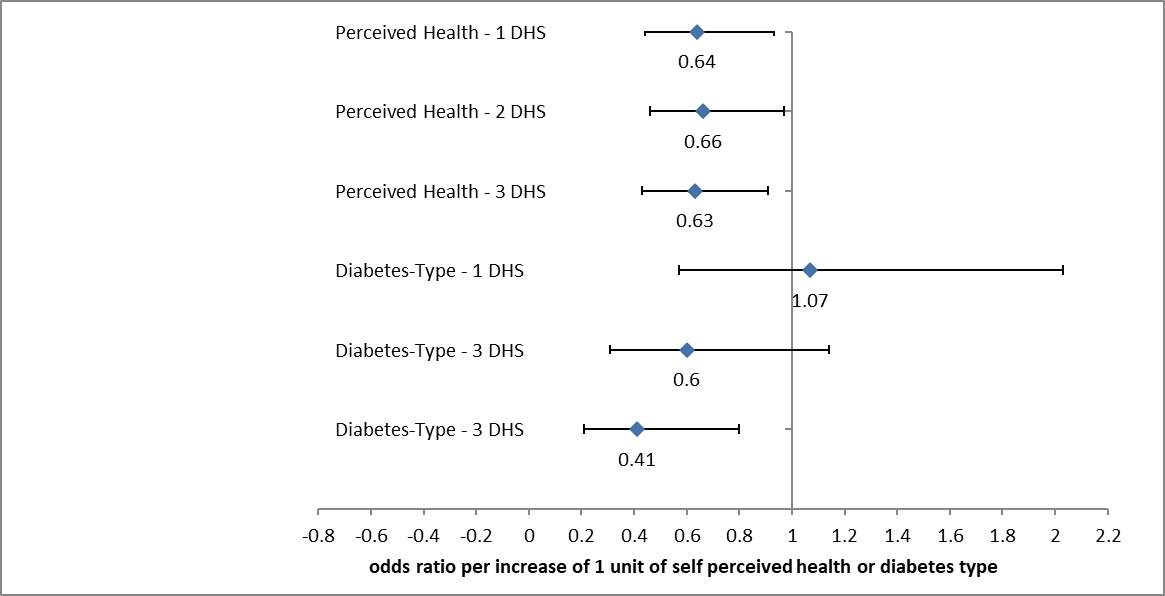


**Supplementary Figure 2.** Results of stepwise logistic regression analyses examining key factors associated with use of different digital health solutions (DHS). In all models, the dependent variable was DHS use status (naïve user vs. experienced user) for the respective DHS. Results are based on of multivariate stepwise logistic regression analysis (shown are odds ratios and their 95% confidence intervals).

- **Figure 2A** shows factors associated with DHS1 use.
- **Figure 2B** shows factors associated with DHS2 use.
- **Figure 2C** shows factors associated with DHS3 use.

The reference category in each model is being a *naïve user* of the respective DHS.

**Independent variables** included sociodemographic and attitudinal factors relevant to each DHS:

- For **DHS1**: diabetes type, perceived improvements in physical and mental health, diabetes management, and access to support; perceived information overload; perceived burden of diabetes demands; self-management failure emphasis; diabetes intrusion into personal life; difficulty trusting information; and anxiety about learning alarming health information.
- For **DHS2**: diabetes type, perceived improvements in physical and mental health, and diabetes management; perceived information overload; perceived burden of diabetes demands; self-management failure emphasis; diabetes intrusion into personal life; concerns about health data storage; and potential recording errors.
- For **DHS3**: diabetes type, perceived improvements in physical and mental health, diabetes management, and support; perceived information overload and diabetes burden; self-management failure emphasis; diabetes intrusion into personal life; fear of judgment based on transmitted data; concerns about data privacy; perception of more impersonal relationships with the diabetes team; perceived cost reduction, better involvement in therapy, improved care coordination, and better treatment focus.

Only variables retained in the final models after stepwise selection are shown. Items are presented in abbreviated form; full item wordings are available in **Table 2**

.

**Supplementary Figure 2A.** Factors associated with DHS1 use.


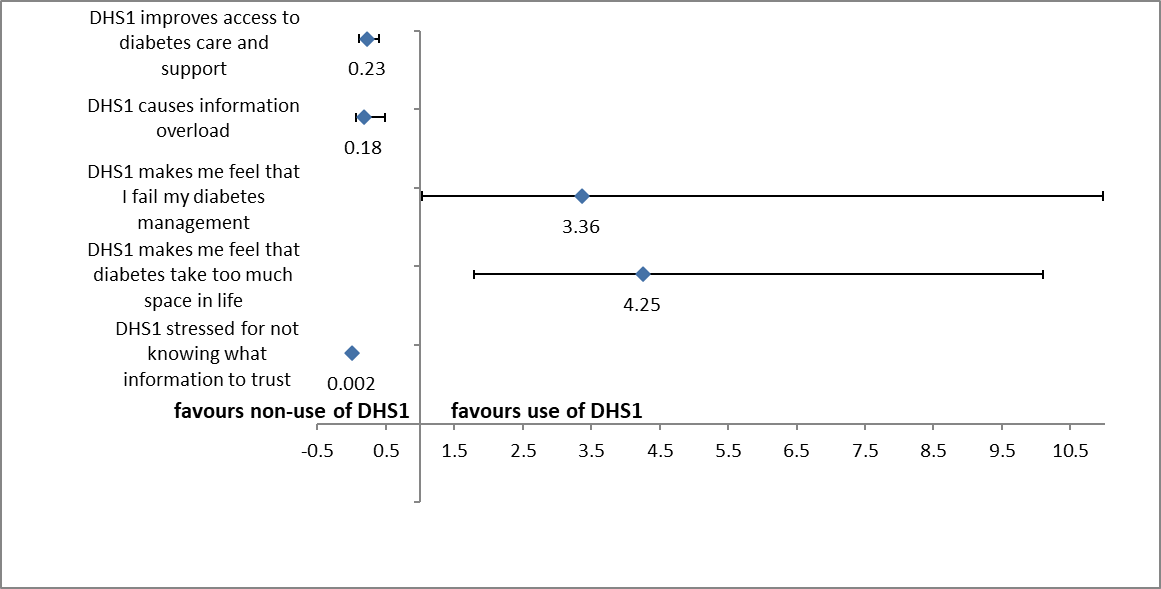


The reference category in each model is being a *naïve user* of the respective DHS.

**Independent variables** included sociodemographic and attitudinal factors relevant to each DHS:

- For **DHS1**: diabetes type, perceived improvements in physical and mental health, diabetes management, and access to support; perceived information overload; perceived burden of diabetes demands; self-management failure emphasis; diabetes intrusion into personal life; difficulty trusting information; and anxiety about learning alarming health information.

**Supplementary Figure 2B.** Factors associated with DHS2 use.


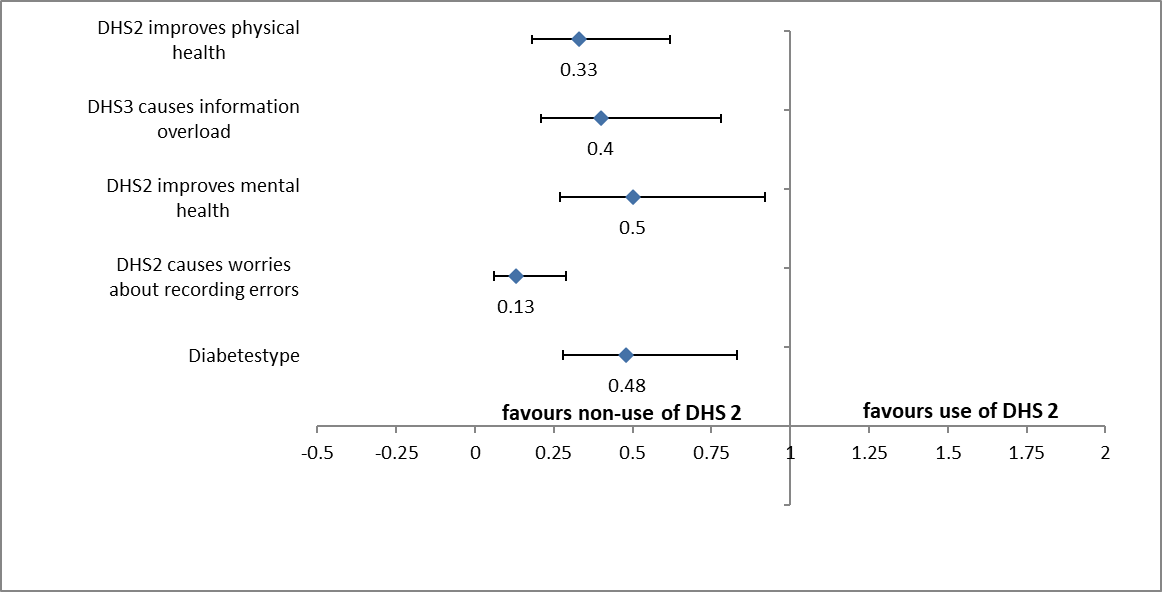


The reference category in each model is being a *naïve user* of the respective DHS.

**Independent variables** included sociodemographic and attitudinal factors relevant to each DHS:

- For **DHS2**: diabetes type, perceived improvements in physical and mental health, and diabetes management; perceived information overload; perceived burden of diabetes demands; self-management failure emphasis; diabetes intrusion into personal life; concerns about health data storage; and potential recording errors.

**Supplementary** **Figure 2C.** Factors associated with DHS3 use.


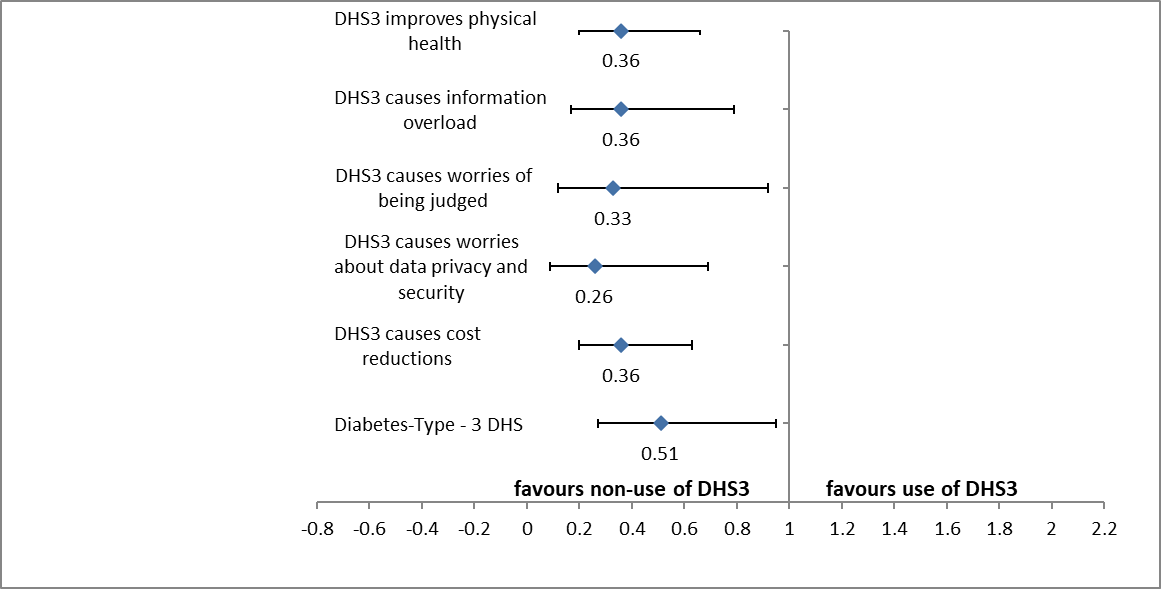


The reference category in each model is being a *naïve user* of the respective DHS.

**Independent variables** included sociodemographic and attitudinal factors relevant to each DHS:

- For **DHS3**: diabetes type, perceived improvements in physical and mental health, diabetes management, and support; perceived information overload and diabetes burden; self-management failure emphasis; diabetes intrusion into personal life; fear of judgment based on transmitted data; concerns about data privacy; perception of more impersonal relationships with the diabetes team; perceived cost reduction, better involvement in therapy, improved care coordination, and better treatment focus.
